# Genome-Wide Analysis of RAV Transcription Factors and Functional Characterization of Anthocyanin-Biosynthesis-Related *RAV* Genes in Pear

**DOI:** 10.3390/ijms22115567

**Published:** 2021-05-25

**Authors:** Jianlong Liu, Zhiwei Deng, Chenglin Liang, Hongwei Sun, Dingli Li, Jiankun Song, Shaoling Zhang, Ran Wang

**Affiliations:** 1 College of Horticulture, Qingdao Agricultural University, Qingdao 266109, China; 201901068@qau.edu.cn (J.L.); zhiweideng@stu.qau.edu.cn (Z.D.); shw@stu.qau.edu.cn (H.S.); lidingli@qau.edu.cn (D.L.); 200601048@qau.edu.cn (J.S.); 2 Haidu College, Qingdao Agricultural University, Laiyang 265200, China; ljl2015lcl@163.com; 3 Centre of Pear Engineering Technology Research, Nanjing Agricultural University, Nanjing 210095, China; nnzsl@njau.edu.cn

**Keywords:** pear, RAV transcription factor, genome-wide analysis, functional characterization, anthocyanin biosynthesis

## Abstract

Related to ABSCISIC ACID INSENSITIVE3/VIVIPAROUS1 (ABI3/VP1, RAV), transcription factors (TFs) belonging to the APETALA2/ETHYLENE RESPONSIVE FACTOR (AP2/ERF) TF family play critical roles in plant growth, development, and responses to abiotic and biotic stress. In this study, 11 novel RAV TFs were identified in pear (*Pyrus bretschneideri* Rehd). A phylogenetic analysis revealed that the TFs clustered into three groups with 10 conserved motifs, some of which were group- or subgroup-specific, implying that they are important for the functions of the RAVs in these clades. RAVs in *Pyrus* and *Malus* were closely related, and the former showed a collinear relationship. Analysis of their expression patterns in different tissues and at various growth stages and their responses to abiotic and biotic stress suggested that *PbRAV6* and *PbRAV7* play important roles in drought stress and salt stress, respectively. We investigated the function of RAVs in pear peel coloration using two red pear varieties with different color patterns and applying data from transcriptome analyses. We found that *PbRAV6* participates in the regulation of pericarp color. These findings provide insight into a new TF family in pear and a basis for further studies on the response to drought stress and fruit coloration in this commercially important crop.

## 1. Introduction

Pear (*Pyrus*) is one of the most important deciduous fruit trees in the world. The planting area of pear in China is 937,642 ha, which accounts for 67.85% of the total area globally [1]. The quality of the fruit is mainly determined by flavor, aroma, and peel color. Red pear varieties are especially popular among consumers. Therefore, the genetic and molecular determinants of pear fruit color are a major focus of research.

Anthocyanins are pigments that confer red, purple, and blue colors to many fruits and vegetables. The key structural genes in the anthocyanin biosynthesis pathway have been extensively studied, and a large number of transcription factors (TFs) that regulate anthocyanin biosynthesis have been identified, such as myeloblastosis (MYB) and basic helix-loop-helix (bHLH). The APETALA2/ETHYLENE RESPONSIVE FACTOR (AP2/ERF) family is a large family of plant TFs that play important roles in the control of primary and secondary metabolism (including anthocyanin biosynthesis), development, and the response to various types of biotic and abiotic stress [2,3]. The AP2/ERF superfamily has been studied in a variety of plants including *Arabidopsis* [4,5], rice [4,5], cotton [6], peanut [7], bamboo [8,9], Chinese plum [10], apple [11], *Eucalyptus grandis* [12], potato [13], musa [14], and pear [15]. AP2/ERF TFs have at least one AP2 DNA-binding domain with a highly conserved sequence of ~60 amino acids [16]. According to the number of repeats and sequence of the AP2 domains, the AP2/ERF family is categorized into the AP2, dehydration-responsive element-binding (DREB), ERF, Related to ABI3/VP1 (RAV), and Soloist subfamilies [17]. AP2/ERF TFs have been found in different species and play important roles in anthocyanin synthesis. For example, *PbMYB114* was shown to interact with *PbERF3* to modulate anthocyanin biosynthesis in pear (*Pyrus bretschneideri* Rehd.) [18]; in *Arabidopsis thaliana*, *AtERF4* and *AtERF8* contributed to light-modulated anthocyanin biosynthesis [19]; and in apple (*Malus domesticus*), *MdERF1B* and *MdERF3* stimulated the biosynthesis of anthocyanin and proanthocyanidin through cooperation with MdMYBs [20,21]. *MdERF1B* not only interacted with *MdMYB11*, but also activated its transcription by binding directly to the gene promoter [20]. Another study showed that *MdERF3* directly activates the expression of MdMYB1 in anthocyanin biosynthesis [21]. Two ERF TFs in Asian pear (*Pyrus pyrifolia*), *Pp4ERF24* and *Pp12ERF96*, were found to stimulate blue-light-induced anthocyanin biosynthesis via interaction with PpMYB114 [22]. As the member of AP2/ERF family, RAVs play an important role in plant development and defense responses [23]. *Arabidopsis* RAV1 and RAV2 were the first identified members of the RAV family [24]; there are 147 AP2/ERF TF genes in *Arabidopsis*, including 18 in the AP2 subfamily (14 with double AP2 domains and 4 with a single AP2 domain), 122 in the ERF subfamily (ERF and DREB/CBF combined), 6 in the RAV subfamily, and a single gene (*AT4g13040*) in the Soloist subfamily [5]. The functions of family members have been studied in different species. In *Arabidopsis* and soybean, RAV1 can regulate seed germination and the regeneration of roots and adventitious buds [25,26]. In tobacco, melon, and cotton, RAV1 can promote plant resistance to drought and high salt stress [27,28,29]. MeRAV1 and MeRAV2 activate melatonin to promote cassava resistance to bacterial blight [30]. Recently, it was found that FaRAV1 can activate MYB10 to promote anthocyanin synthesis in strawberry [31]. Although RAV TFs are known to be involved in physiological and biochemical processes in plants, the RAV TFs of *Pyrus* have not been identified and characterized, including in terms of their expression at different stages of pear fruit development.

To address this issue, we carried out a genome-wide analysis of Chinese white pear (*P. bretschneideri*) RAV genes and investigated their potential functions in anthocyanin biosynthesis and plant responses to stress using the ‘Xinli No. 7’ cultivar and its red bud variety ‘Xinqihong’.

## 2. Results

### 2.1. Identification and Phylogenetic Analysis of RAV TFs in Pear

Putative pear RAV sequences were identified by searching the Rosaceae genome using the Basic Local Alignment Search Tool for protein sequences (BLASTP). Eleven RAV protein sequences were screened based on domain and were renamed *PbRAV1*–*PbRAV11* according to chromosome sequence and location (Table 1). The polypeptide lengths of the predicted pear RAVs ranged from 303 to 380 amino acids. The predicted subcellular localization was the nucleus for PbRAV1, PbRAV5, and PbRAV7, and the cytoplasm for the other RAVs. The molecular weight, isoelectric point (pI), and grand average of hydropathicity (GRAVY) are also summarized in Table 1.

**Figure 1:**
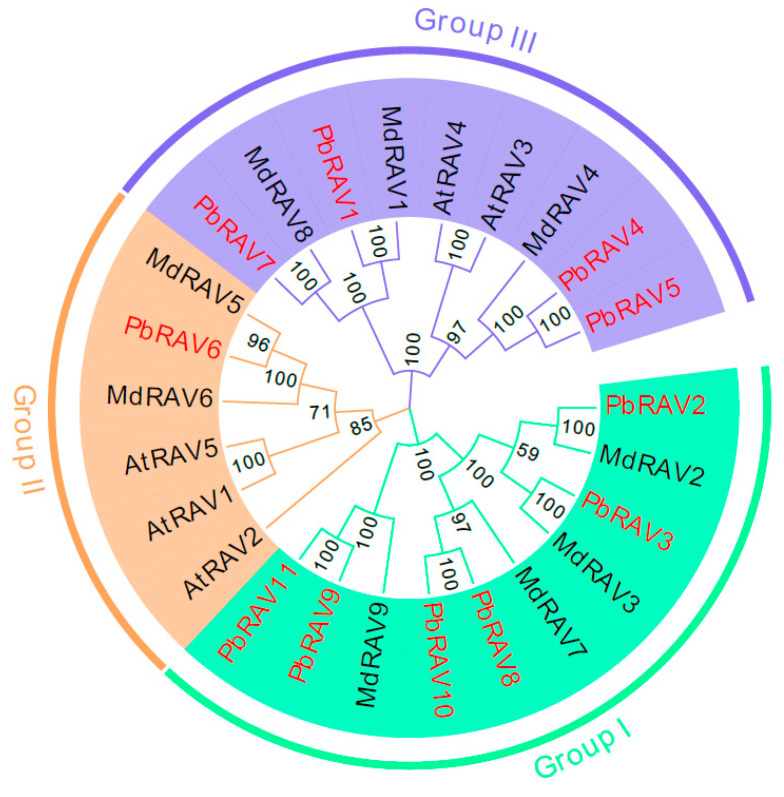
Phylogenetic tree constructed with the neighbor-joining method using RAV TFs domains in *P. bretschneideri*, *M. domestica*, and *Arabidopsis*. The tree was divided into three groups (Groups I, II, and III).

We examined the phylogenetic relationships among pear RAVs by generating a phylogenetic tree using the neighbor-joining method. The tree included 11 *P. bretschneideri*, 9 *M. domestica*, and 5 *Arabidopsis* RAV TFs (Figure 1). The family was divided into three groups (Figure 1). PbRAV2, PbRAV3, PbRAV8, PbRAV9, PbRAV10, and PbRAV11 were more closely related to MdRAV2, MdRAV3, MdRAV7, and MdRAV9 and were separate from the RAVs in *Arabidopsis*. PbRAV6 was in group II, while the other PbRAVs were in group I or III.

### 2.2. Conserved Motifs and Gene Structure of PbRAV TFs

**Figure 2:**
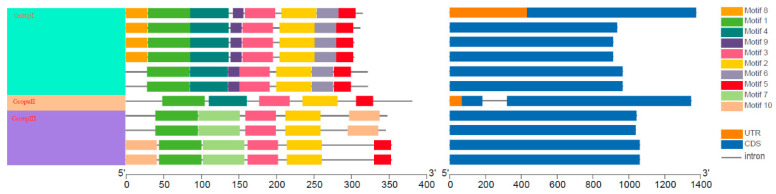
Schematic representation of protein and gene structures of *P. bretschneideri RAV* genes. Motifs 1–10 identified using the MEME search tool are marked on the protein sequences in each clade (I–III). The length and order of each motif corresponds to the actual length and position in the protein sequences. The coding sequence and untranslated regions are represented by filled dark blue and orange boxes, respectively.

The conserved motifs of *PbRAV*s were analyzed using Multiple Em for Motif Elicitation (MEME). Ten conserved motifs were predicted in *PbRAV*s (Figure 2 and Supplementary Figure S1); their sizes ranged from 27 to 112 amino acids. Motifs 1, 2, and 3 were present in all *PbRAV*s; motifs 6, 8, and 9 were found in members of group I; motifs 10 and 7 were present in members of group III; motifs 4 and 5 were present in members of groups I and II; and motif 5 was found in *PbRAV7* and *PbRAV8*. The majority of *PbRAV* genes had a single exon and only one (*PbRAV6*) had introns (Figure 2).

### 2.3. Chromosomal Distribution and Orthologous Relationships of PbRAV Genes in P. bretschneideri, Arabidopsis, and M. domestica

**Figure 3:**
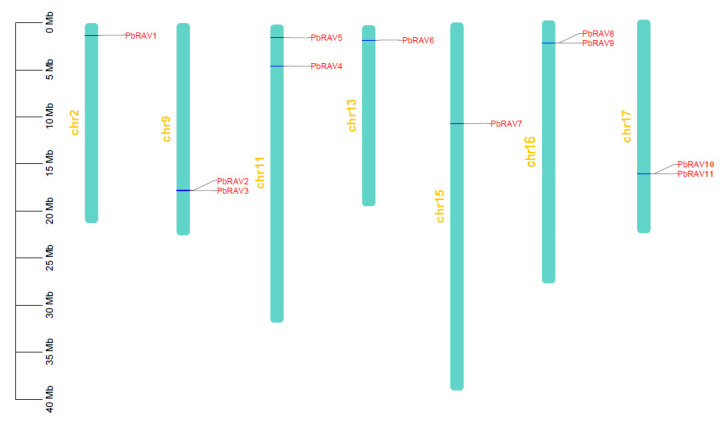
Chromosomal distribution of *PbRAV* genes drawn using MapChart v2.2. Scale represents chromosome length: 0–40 Mb.

**Figure 4:**
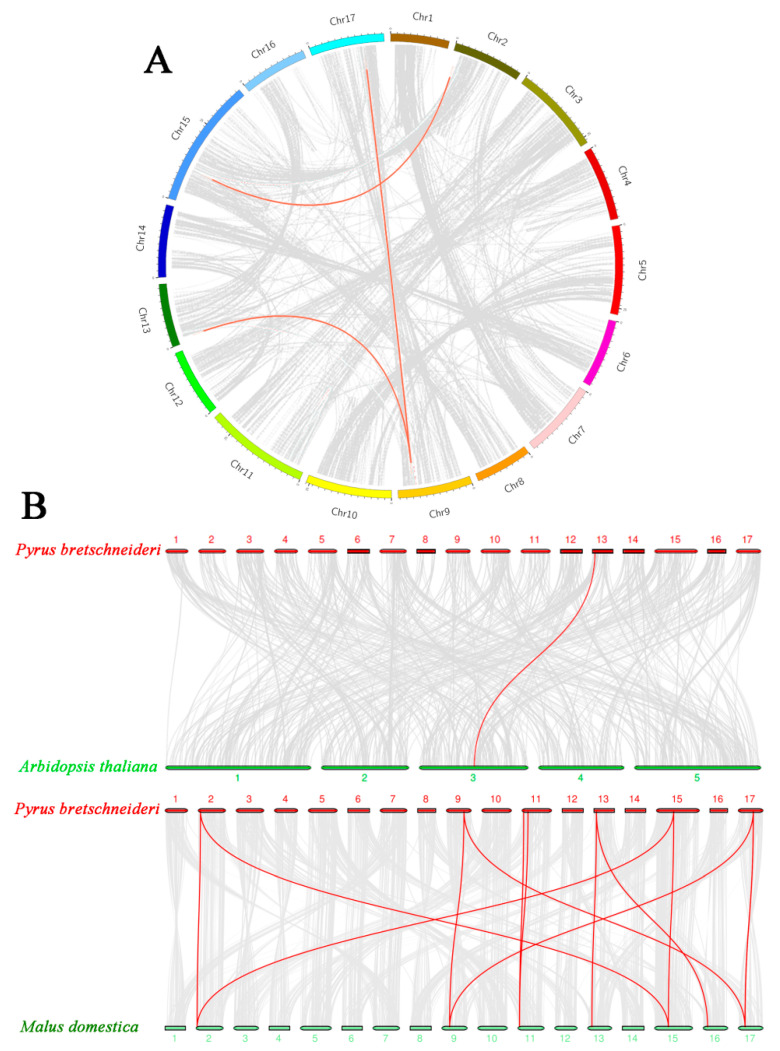
Collinearity of RAVs. (**A**) Distribution of segmentally duplicated *PbRAV* genes on *P. bretschneideri* chromosomes. Gray lines indicate collinear blocks in the whole *P. bretschneideri* genome and red lines indicate duplicated *PbRAV* gene pairs. (**B**) Collinearity of *P. bretschneideri*, *Arabidopsis*, and *M. domestica* RAV genes. Red lines indicate the syntenic gene pairs.

Physical mapping of *PbRAV*s on the 17 chromosomes of *P. bretschneideri* revealed an uneven distribution (Figure 1); the genes were distributed on just 7 chromosomes, with one or two per chromosome. The exact position (in bp) of each *PbRAV* is presented in Table 1. Two *PbRAV* genes were tandem repeats (Figure 3) and five (three pairs) were segmentally duplicated (Figure 4A). To establish the orthologous relationships of *PbRAV*s, we compared the physical location of *RAV* genes in the genomes of *P. bretschneideri*, *A. thaliana*, and *M. domestica* (Figure 4B). Only *PbRAV6* showed a close evolutionary relationship to the *RAV* genes in *Arabidopsis*. On the other hand, RAVs in *Malus* and *Pyrus* showed a close collinear relationship.

### 2.4. Expression Pattern of RAV Genes during Pear Development

**Figure 5:**
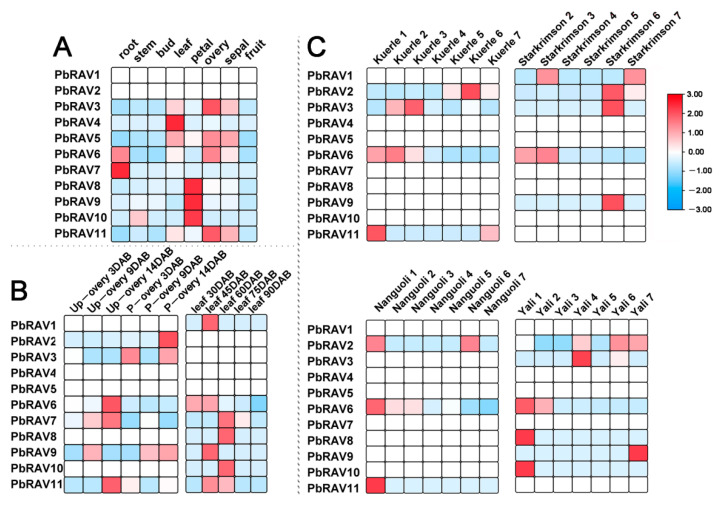
Expression profiles of *PbRAV*s in pear. (**A**,**B**) Expression profile of *PbRAV*s in different tissues (**A**) and in ovary and leaf at different development stages (**B**) of *P. bretschneideri* ‘Xinli No. 7.’ (**C**) Expression profile of *PbRAV*s at different fruit development stages in various *P. bretschneideri* varieties. P-ovary: pollinated ovary; Up-ovary: unpollinated ovary.

We analyzed the spatiotemporal expression patterns of *PbRAV* genes in eight different tissues and at different developmental stages using publicly available gene expression data. Of the examined *PbRAV* genes, 2 were not expressed in any of the tissue types, 1 was expressed in all eight tissue types and was constitutively expressed (fragments per kilobase of transcript per million mapped reads (FPKM) > 10), and 10 were differentially expressed across tissues (Figure 5). Although 7 genes were expressed during ovary development, 6 of these had low abundance (FPKM < 2) and only *PbRAV6* had high expression. The abundance of *PbRAV6* was higher during the development of leaf and four representative fruits, and gradually decreased with developmental stage. It is worth noting that the expression of *RAV*s was not consistent among different varieties of pear: only 3 genes were expressed in ‘Nanguo’ while 6 were detected in ‘Yali’.

### 2.5. Expression of PbRAV Genes in Response to Abiotic and Biotic Stress

**Figure 6:**
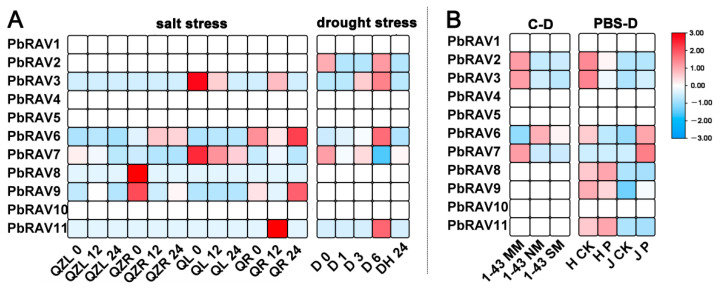
Expression profiles of *PbRAV* genes under abiotic and biotic stress. (**A**) Abiotic stress included salt and drought stress. (**B**) Biotic stress included cork spot and pear black spot disease. C-D: cork spot; MM: moderate disease mesocarp; NM: normal mesocarp; PBS-D: pear black spot disease; QL: ‘Qaup-1’ leaf, QR: ‘Qaup-1’ root; QZL: ‘Qingzhen D1’ leaf; QZR: ‘Qingzhen D1’ root; SM: severe disease mesocarp.

To determine whether *PbRAV* gene expression was affected by different types of stress, we analyzed the genes’ expression patterns following exposure to abiotic and biotic stress (Figure 6). Four of the *PbRAV*s (*PbRAV1*, *PbRAV4*, *PbRAV5*, and *PbRAV10*) were not expressed under the different treatments. Under salt stress, although *PbRAV6* had higher expression abundance, the expression changed irregularly. However, *PbRAV7* expression decreased with the increase of salt damage time in two varieties. Under drought stress, *PbRAV6* was upregulated as drought duration increased but was downregulated after exposure to water. In fruit samples with cork spot, *RAV* gene expression was low, which may have been due to the fact that the samples were fruit. In addition to *PbRAV6*, *PbRAV8* and *PbRAV9* were expressed in all samples exposed to biotic stress.

### 2.6. Expression of PbRAV Genes during Anthocyanin Biosynthesis

**Figure 7:**
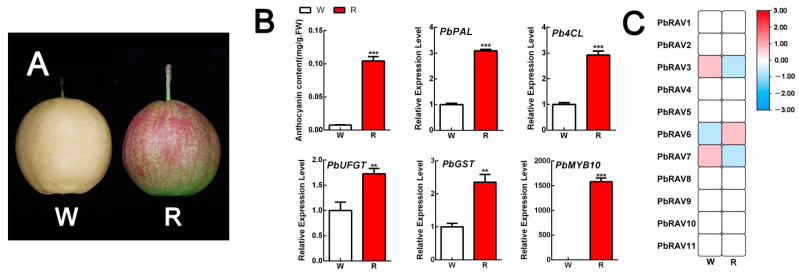
Expression profile of *PbRAV*s in red pear peel. (**A**) Appearance of ‘Xinqihong’ pear after bagging. (**B**) Anthocyanin content and expression of genes regulating anthocyanin biosynthesis. (**C**) Expression profile of *PbRAV*s in white and red pear peel. ** *p* < 0.01, *** *p* < 0.001 (Student’s *t* test). R: red pear peel; W: white pear peel. The expression levels of genes in the peel of white pear under control conditions were normalized as 1.0.

**Figure 8:**
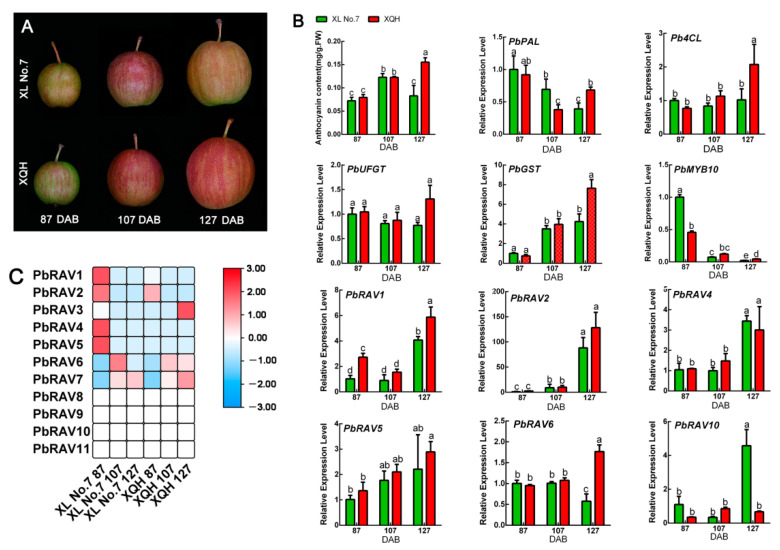
Expression profiles of *PbRAV*s in two pear cultivars during the fading period. (**A**) Morphological changes in ‘Xinli No. 7’ and ‘Xinqihong’ pear varieties during the fading period. (**B**) Anthocyanin content, expression of genes regulating anthocyanin biosynthesis, and expression of PbRAVs. (**C**) Expression profile of *PbRAV*s in the two pear cultivars. Different lowercase letters indicate statistical significance at *p* < 0.05 (Duncan’s test). The expression levels of genes in the peel of ‘Xinli No. 7’ pear at 87 DAB were normalized as 1.0. DAB: days after full bloom.

To investigate the role of *PbRAV*s in anthocyanin biosynthesis, we prevented anthocyanin production in the red pear cultivar ‘Xinqihong’ by bagging the fruit, which resulted in the fruit peel changing to a white color (Figure 7). Anthocyanin content and the expression of related genes were markedly higher in red than in white fruit. Only three *PbRAV*s were expressed, but the abundance of *PbRAV3* and *PbRAV7* was low (FPKM < 1); in contrast, *PbRAV6* was highly expressed and the level was higher in red pear than in white pear. The qRT-PCR results were consistent with the transcriptome analysis (Figure S2). We further analyzed the ‘Xinli No. 7’ and ‘Xinqihong’ pear varieties (Figure 8). The latter is the red bud mutation of the former and remains red at the late fruit stage, whereas the red color of ‘Xinli No. 7’ fades at the late fruit stage. We found that the red color of ‘Xinqihong’ at the late fruit stage was due to a higher anthocyanin content, with the expression pattern of *PbRAV6* reflecting the color change. Among them, PbRAVs with low expression abundance were not verified quantitatively.

## 3. Discussion

As one of the largest gene families in plants, the AP2/ERF family plays a critical role in multiple physiological and biochemical processes by regulating the expression of genes involved in the response to various stressors in *Arabidopsis*, *Taxus chinensis* [32], cotton [33], and desert moss (*Bryum argenteum*) [15]. Although this family has been extensively studied, there have been few reports to date on the RAV subfamily; in particular, there is limited information on the regulation and structure of these genes in pear. In this study, we analyzed whole-genome data of *P. bretschneideri* and identified genes encoding RAV family TFs, explored their roles in growth development and response to stress based on published expression data of *Pyrus* tissues, and explored their roles in anthocyanin biosynthesis between pear varieties with a significant color difference.

A total of 11 RAV genes were identified in *P. bretschneideri* and were classified into three families based on conserved domains. *PbRAV2*, *PbRAV3*, *PbRAV8*, *PbRAV9*, *PbRAV10*, and *PbRAV11* in pear were grouped with *MdRAV2*, *MdRAV3*, *MdRAV7*, and *MdRAV9* in apple, suggesting that *RAV* genes in pear and apple emerged together during evolution and share a common ancestor. The number of species harboring *RAV* superfamily genes in *Pyrus* was higher than the numbers in *Arabidopsis* (5) and *Malus* (9). This may be the result of gene duplication in plants, which is considered a fundamental driving force in the evolution of genomes [34] as it provides raw material for new genes that can then lead to the emergence of new functions. Segmental duplication, tandem duplication, and transposition events such as retro- and replicative transposition [35] are the three main patterns of gene evolution; the first two are thought to underlie gene family expansion in plants [36]. Tandem duplications can be identified based on the presence of multiple members of a single gene family within the same or in neighboring intergenic regions [37].

Among the 11 *RAV* genes in pear, there were two pairs of tandem repeats (*PbRAV2*/*PbRAV3* and *PbRAV8*/*PbRAV9*). We also detected three segmental duplication pairs involving five *PbRAV* genes (*PbRAV2*/*PbRAV8*, *PbRAV2*/*PbRAV6*, and *PbRAV1*/*PbRAV7*), suggesting that segmental duplication was mainly responsible for expansion of the *RAV* gene family in *Pyrus*. A syntenic analysis of *RAV* genes in *Pyrus*, *Arabidopsis*, and *Malus* showed that *PbRAV* genes had higher homology with *RAV* genes in *Malus* and lower homology with those in *Arabidopsis*. However, *PbRAV6* was related to *Arabidopsis* RAVs, indicating that RAV6 in different plants may have evolved from a common ancestor.

Conserved domains or amino acid motifs in TFs are frequently involved in DNA binding, nuclear localization, protein–protein interactions, and transcriptional activity [10], and TFs with similar domains or motifs are likely to have similar functions. We found that all *PbRAV* members harbored motifs 1, 2, and 3. All members of group I had motifs 6 and 9, which were not found in other group members, while motif 10 was only present in group III. The distribution of these motifs may account for the variable responses of *PbRAV*s to different conditions.

Introns not only function in the regulation of gene expression, but also participate in gene evolution [38]; this has been observed in mammals, nematodes, insects, fungi, and plants. Our analysis of *RAV* gene structure revealed that one of genes (*PbRAV6*) contained a single intron, whereas the others had no introns. *PbRAV* genes were expressed in different plant tissues under normal conditions, with *PbRAV6* being the most highly expressed. Thus, *RAV* gene expression may be affected by the presence or absence of an intron. Moreover, the abundance of *PbRAV*s varied across tissues and *PbRAV*s showed distinct expression profiles, implying that these genes have specific roles in different pear tissues. For example, *PbRAV3* and *PbRAV5* were highly expressed in leaf, petal, and ovary but not in root, whereas *PbRAV6* was highly expressed in root, leaf, ovary, and sepal. On the other hand, the expression of *PbRAV1*, *PbRAV2*, and *PbRAV10* was low in all tissues, suggesting that these genes are not involved in plant development. Indeed, *PbRAV6* expression decreased as development progressed—including in leaf and fruit—whereas *PbRAV2* was upregulated. The fact that the downregulation of *PbRAV6* also occurred during pollination confirms the important role of this gene in development.

Unlike animals, plants must adapt to various biotic and abiotic stressors throughout their life cycle. This requires the activation of genes associated with a resistant phenotype by TFs [39]. In our study, *PbRAV*s were expressed at low levels in fruit under biotic and abiotic stress. In diseased fruit, only *PbRAV6* was present in all tissues. *PbRAV6* and *PbRAV7* were both expressed under drought stress; *PbRAV6* expression increased with drought duration but decreased after watering. This indicates that PbRAV6 was involved in the response of pear to drought stress and was closely related to plant water deficit. On the other hand, *PbRAV6* level decreased with a longer duration of salt stress, but the pattern was irregular. However, the expression of *PbRAV7* decreased with the increase of salt stress time, indicating that *PbRAV7* was negatively responsive to salt stress. Thus, different *PbRAV* genes show distinct responses depending on the type of stressor, which may be related to their structural diversity.

It is worth noting that PbRAVs play an important role in the regulation of peel color. Although their levels in fruit were low overall, there was a significant difference in the abundance of PbRAV6 in white vs. red peel, and it was always highly expressed in red peel. Furthermore, in ‘Xinli No. 7’ and ‘Xinqihong’ (the red bud mutation of ‘Xinli No. 7’), the expression level of *PbRAV6* was same at the early coloring stage, but the expression level of *PbRAV6* in ‘Xinli No. 7’ after fading was significantly lower than that in ‘Xinqihong’ at the late coloring stage, which indicates that the expression of *PbRAV6* was closely related to the synthesis of anthocyanins. In strawberry (*Fragaria ananassa*), FaRAV1 was shown to promote anthocyanin accumulation by directly binding to the promoter of various genes in the anthocyanin biosynthesis pathway and increasing their expression including FaMYB10 (4.0 fold), chalcone synthase (CHS; 1.53-fold), flavanone hydroxylase (F3H; 1.95-fold), dihydroflavonol 4-reductase (DFR; 3.6-fold), and 5,3-O-glucosyltransferase (GT)1 (2.3-fold) [40]. In pear, PbRAV6 may have a similar function, which requires further investigation.

## 4. Materials and Methods

### 4.1. Database Search and Sequence Retrieval

The complete genome assembly of pear (*P. bretschneideri* Rehd) along with the complete proteome sequence file was downloaded from the Genome Database for Rosaceae (https://www.rosaceae.org (accessed on 5 March 2021)). Conserved amino acid sequences were used as a query to identify RAV TFs in the *Pyrus* proteome sequence file using CLC Sequence Viewer v7.6.1 [31]. Putative RAV TFs were confirmed by BLASTP searches of the NCBI database. This database was also used to obtain gene accession numbers, chromosome number, genomic information, and the protein size of RAV TFs. The nucleotide sequences of all identified RAVs were also retrieved from NCBI. The ExPASY online tool (https://web.expasy.org/protparam (accessed on 12 March 2021)) was used to calculate molecular weight, pI, and GRAVY of all PbRAV proteins. WoLF PSORT (http://www.genscript.com/psort/wolf_psort.html (accessed on 12 March 2021)) was used to predict the subcellular localization of PbRAV proteins.

### 4.2. Chromosomal Mapping, Intron/Exon Distribution, and Conserved Domain Analysis

The NCBI database was used to record the positions of the identified *PbRAV* genes on chromosomes, and Map Chart v2.32 was used to draw a map of chromosomal locations of *PbRAV* genes to scale [41]. Gene Structure Display Server (v2.0 http://gsds.cbi.pku.edu.cn/ (accessed on 15 March 2021)) was used to draw and visualize the intron–exon organization of *RAV* genes [42]. The whole genome sequence and the coding sequences of all *RAV* genes were used to construct the gene structure map containing introns.

In order to identify domains conserved among all *PbRAV*s, their protein sequences were analyzed with MEME v5.0.3 [43] using default parameters, except that the minimum number of motif sites was set to 10.

### 4.3. Phylogenetic Comparison of RAV Proteins in P. bretschneideri, M. domestica, and A. thaliana

A phylogenetic tree was constructed using the protein sequences of putative RAV TFs from *P. bretschneideri* and *M. domestica*, with *A. thaliana* sequences used as a reference. Multiple sequence alignment of the 11 PbRAV proteins was performed using ClustalW v1.83. A total of 25 plant RAV proteins were used to construct the phylogenetic tree, including 11 from pear, 9 from apple, and 5 from *Arabidosis*. The tree was used to infer the evolutionary history and functions of PbRAV TFs. Their coding sequences were used for pairwise alignment with the built-in ClustalW and PAM protein weight matrix of MEGA7 [44]. The resultant alignments were analyzed for DNA sequence polymorphisms and DnaSP v5.10.01 [45] was used to compute the synonymous substitution rate (Ks) and nonsynonymous substitution rate (Ka). Ks/Ka was also calculated to evaluate codon selection during evolution.

### 4.4. Expression Patterns of PbRAVs in Various Tissues of Pear at Different Developmental Stages

The expression patterns of *PbRAV* genes in various tissues at different developmental stages were obtained from the pear gene expression atlas. The raw sequence reads from seven pear tissues were analyzed using the NCBI web browser (https://www.ncbi.nlm.nih.gov/bioproject/; accession no. PRJNA498777 (accessed on 12 March 2021)) [46]. The expression patterns of *PbRAV* genes at three different stages of ovary development and under two treatments (unpollinated (control) and hand-pollinated) were analyzed using 15 year old ‘Dangshansuli’ trees grafted onto *P. betulifolia* Bge rootstocks; three branches per treatment were used as three replicates. Ovaries were collected at 3, 9, and 14 days after full bloom (DAB) [47]. For the analysis of expression patterns of *PbRAV* genes in leaf samples at different developmental stages of pear, leaves were collected at 30, 45, 60, 75, and 90 DAB; the RNA sequencing (RNA-seq) data were downloaded from the Sequence Read Archive (SRA) (accession nos. SRR10997902–SRR10997912) [48]. Finally, the expression profiles of *PbRAV* genes based on RNA-seq data from four pear varieties at seven fruit development stages were downloaded from the SRA (accession no. SRP070620). The transcriptomes of four representative cultivars—namely ‘Yali’ (*P. bretschneideri*), ‘Kuerlexiangli’ (*P. sinkiangensis*), ‘Nanguoli’ (*P. ussuriensis*), and ‘Starkrimson’ (*P. communis*), which are the main cultivated species worldwide—were used for the analysis of gene expression patterns and to explore the relationship between different fruit traits. The following fruit development stages were analyzed: fruit setting (period 1), physiologic fruit dropping (period 2), rapid fruit enlargement (period 3), 1 month after fruit enlargement (period 4), pre-maturity (period 5), and maturity (period 6); additionally, one fruit senescence stage after harvest (period 7) was included in the analysis [49].

### 4.5. Expression Patterns of PbRAVs under Different Stress Conditions

Expression patterns of *PbRAV*s in response to abiotic and biotic stress were based on microarray data downloaded from the SRA (series matrix accession no. SRP051914 and SRP148620) [50,51] and data from the present study.

#### 4.5.1. Salt Stress

This experiment was conducted in the pear breeding laboratory of Qingdao Agricultural University. Two varieties of pear, ‘QAUP-1’ (*P. ussuriensis* Maxim) and ‘Qingzhen D1’ (*P. communis* L. × *P. bretschneideri* Rehd), were used. Plants of similar size (5–6 leaves, about 10 cm tall) were selected after 40 days of growth and transferred to plastic tubs (50 cm × 35 cm × 15 cm) containing 20 L of a half-strength Hoagland’s nutrient solution [52]. The tubs, wrapped with black plastic to block light exposure to the root systems, were placed in a growth chamber (25 °C/18 °C day/night). Light was provided by sodium lamps during a 14 h photoperiod (photon flux density of 150 μmol/m^2^/s). The nutrient solution was continuously aerated with an air pump, and dissolved oxygen concentrations were maintained at 8.0–8.5 mg/L by a dissolved oxygen controller (FC-680; Corporation of Super, Shanghai, China). The pH was adjusted to 6.5 ± 0.1 with H_3_PO_4_, and the solution was refreshed every 3 days. Seedlings were precultured for 10 days to allow them to adapt to the new conditions. After adaption to the culture environment, salt stress treatment was applied: half-strength nutrient solution and 100 mmol/L NaCl. Leaf and root samples were collected from the plants 0, 12, and 24 h after treatment along with control samples. Each treatment contained three replicates of 50 plants. The materials were stored at −80 °C, and then used for transcriptional sequencing.

#### 4.5.2. Drought Stress

*P. betulaefolia* plants used in this experiment were 3 months old. Uniform and healthy plants were placed in a beaker containing distilled water that was kept in a growth chamber at 26 °C with a 16 h light/8 h dark photoperiod for 2 days before dehydration treatment. Seedlings were transferred to clean filter paper (90 mm × 90 mm) and allowed to dry for 0, 1, 3, and 6 h at 26 °C, followed by recovery in water at 26 °C for 24 h. The leaves were independently harvested at the designated dehydration time points, immediately placed in liquid nitrogen, and stored at −80 °C until they were used for RNA extraction. Each treatment contained three biological repeats [50].

#### 4.5.3. Cork Spots

Fruits of ‘1–43’ pear were obtained from the Jiaozhou experiment and demonstration station of Qingdao Agricultural University in Qingdao, Shandong Province, China. The superior pear line ‘1–43’ is the hybrid from ‘Xinli No. 7’ (*P. bretschneideri* Rehd) × ‘Zhongxiang Pear’ (*P. bretschneideri* Rehd). Its fruit showed cork spots at 180 days after anthesis. We took normal pear flesh without disease, pear flesh with moderate disease and pear flesh with serious cork spots 180 days after flowering as materials, including three biological repeats. The materials were stored at −80 °C, and then used for transcriptional sequencing.

#### 4.5.4. Pear Black Spot Disease

For the inoculations, inoculum production was conducted using a pear black spot (PBS) strain (H) obtained from leaves of the ‘Xiangnan’ pear from the germplasm bank of Wu Chang Sand Pear Garden National Fruit-tree Germplasm Resource (WCSPGNFGR). The H strain can infect the ‘Hongfen’ pear but not the ‘Jinjing’ pear. For inoculation assays with PBS, the concentration of conidia was adjusted to 1 × 10^6^ spores mL^−1^. The spore suspension was sprayed onto detached young leaves of ‘Jinjing’ and ‘Hongfen’ pear varieties with a glass atomizer. Control leaves were sprayed with distilled water. The inoculated leaves were incubated in a humid chamber at 28 °C. Samples H-CK, H-P, J-CK, and J-P were used in the experimental design and data analysis. All of the samples were tested in triplicate, and the experiments were performed on three biological replicates [51].

### 4.6. Plant Growth Conditions and Quantitative Real-Time (qRT)-PCR Analysis

Samples of healthy and uniform 3 year old ‘Xinqihong’ and ‘Xinli No. 7’ pear plants that had been grafted on *P. betulifolia* Bunge rootstock were collected at Jiaodong Peninsula Regional Experimental Park (37.52° N, 120.25° E), which is in a region with a warm temperate continental monsoon climate, an average annual precipitation of 672.5 mm, and an average annual temperature of 12.6 °C. ‘Xinqihong’ pears were bagged (white), while others were not bagged (red) as controls. The peel was harvested at the commercial ripening stage for transcriptome analysis. ‘Xinli No. 7’ (*P. bretschneideri* Rehd.) red pear cultivar and its bud spontaneous mutation ‘Xinqihong’, which has fruits with a stronger red color, were collected and the pericarp was obtained for transcriptomic analysis during the normal growth periods of ‘Xinli No. 7’ (coloring, 87 DAB; coloring peak, 107 DAB; and fading, 127 DAB). Three replicate plants were analyzed for each time point.

Total RNA of pear peel samples at the three different stages was extracted using the RNAprep Pure Plant Plus Kit (Tiangen, Beijing, China) and treated with DNase I (RNase-free; Takara Bio, Dalian, China) to eliminate residual contaminating genomic DNA. For qRT-PCR, 1.5 μg total RNA was used to synthesize first-strand cDNA with the PrimeScript II 1st Strand cDNA Synthesis kit (Takara Bio) according to the manufacturer’s instructions. qRT-PCR amplification was carried out as follows: 95 °C for 5 min, 45 cycles at 95 °C for 15 s, 60 °C for 30 s, and 72 °C for 30 s using the Roche 480 real-time PCR system (Basil, Switzerland) in standard mode with the FastStart Essential DNA Green Master kit. All reactions were performed in triplicate at a volume of 20 µL, containing 2 µL of 10-fold diluted cDNA. The *Pyrus ACTIN* gene was used as the reference to normalize the qRT-PCR data, and relative gene expression levels were determined via the 2^−ΔΔCT^ method [53]. All experiments were performed with three biological replicates and all primer sequences are shown in Supplementary Table S1.

### 4.7. Measurement of Anthocyanin Content in Peel

Approximately 1 g of fruit peel was ground to a fine powder in liquid nitrogen and extracted with 5 mL of extraction solution (1% HCl in methanol) at 4 °C for 12 h. After centrifugation at 12,000× *g* for 20 min, the supernatant was transferred to a clean tube and the absorbance at 510 nm was measured with a spectrophotometer (UV1800; Meipuda, Shanghai, China). The anthocyanin content was calculated using the equation Ca = 1000 × A × V/(a × b × W), where Ca is the total anthocyanin content (mg/g), A is the absorbance value, V is the extraction solution volume, a is the absorptivity of anthocyanin (0.0775), b is the thickness of the colorimetric ware, and W is the fresh weight of fruit skin [54].

### 4.8. Statistical Analysis

Statistical analysis was performed using Excel 2020 (Microsoft, Redmond, WA, USA). Values are represented as the mean ± SD of three independent biological replicates. Data were analyzed with Duncan’s test, and *p* ≤ 0.05 was considered significant.

## 5. Conclusions

In this study, 11 RAV TFs from pear were identified and characterized based on genome sequences. The phylogenetic analysis of *PbRAV*s provided insight into the evolution of RAV family TFs in fruit plants. Based on our analysis of their expression patterns in pear tissues at different growth stages, we speculate that *PbRAV6* and *PbRAV7* play an important role in the responses to drought and salt stress, respectively; moreover, the identified expression patterns of pear peel with different coloring patterns showed that *PbRAV6* is involved in the regulation of fruit peel color. Although additional studies are needed to clarify the functions of these genes, our findings can provide valuable resources to better understand the biological roles of *RAV* genes in pear, and serve as a valuable resource for future investigations on the functions of RAV TFs in fruit plants and the genetic and molecular mechanisms underlying fruit color.

## Figures and Tables

**Table 1:** General information on *Pyrus bretschneideri RAV* genes.

Gene Name	Gene ID Number ^1^	Chromosome	Start Site	Termination Site	Amino Acid Residues	MW (Da)	pI	Hydrophilicity	Subcellular Localization ^2^
PbRAV1	rna20334	2	1254332	1255369	345	39,656.68	8.75	−0.663	Nuclear
PbRAV2	rna11028	9	17713298	17714715	314	35,670.50	8.61	−0.525	Cytoplasmic
PbRAV3	rna11030	9	17751768	17752703	311	35,287.23	8.98	−0.497	Cytoplasmic
PbRAV4	rna18153	11	4367928	4368989	353	40,901.03	6.34	−0.635	Cytoplasmic
PbRAV5	rna51022	11	1362857	1363918	353	40,901.03	6.34	−0.635	Nuclear
PbRAV6	rna37631	13	1545457	1547183	380	41,708.85	9.14	−0.553	Cytoplasmic
PbRAV7	rna12487	15	10731607	10732650	347	40,063.17	8.87	−0.631	Nuclear
PbRAV8	rna50871	16	2581667	2582926	313	35,191.35	8.79	−0.491	Cytoplasmic
PbRAV9	rna50870	16	2568673	2569996	331	37,772.82	8.42	−0.568	Cytoplasmic
PbRAV10	rna25443	17	16315324	16316296	303	34,391.24	8.89	−0.491	Cytoplasmic
PbRAV11	rna25444	17	16328353	16329416	321	36,732.82	8.71	−0.568	Cytoplasmic

^1^ From *Pyrus bretschneideri* Genome Sequence Consortium database. ^2^ Predicted using WoLFPSORT (http://www.genscript.com/psort/wolf_psort.html (accessed on 12 March 2021)). Abbreviations: MW: molecular weight; pI: theoretical isoelectric point.

## Data Availability

Data is contained within the article or supplementary material.

## Supplementary Materials

The following are available online at https://www.mdpi.com/article/10.3390/ijms22115567/s1, Figure S1: Protein sequences of conserved domains in RAVs, Figure S2: Expression patterns of PbRAVs genes between white and red peel, Table S1: List of qRT-PCR Primers.
