# Supplementary material for: Genome-Wide Analysis of RAV Transcription Factors and Functional Characterization of Anthocyanin-Biosynthesis-Related RAV Genes in Pear
Source: Int J Mol Sci. 2021 May 25;22(11):5567. doi: 10.3390/ijms22115567 (PMC8197526; doi:10.3390/ijms22115567)
Supplement: Supplementary file 1 [file ijms-22-05567-s001.zip › fig S.pdf]

Figure S1

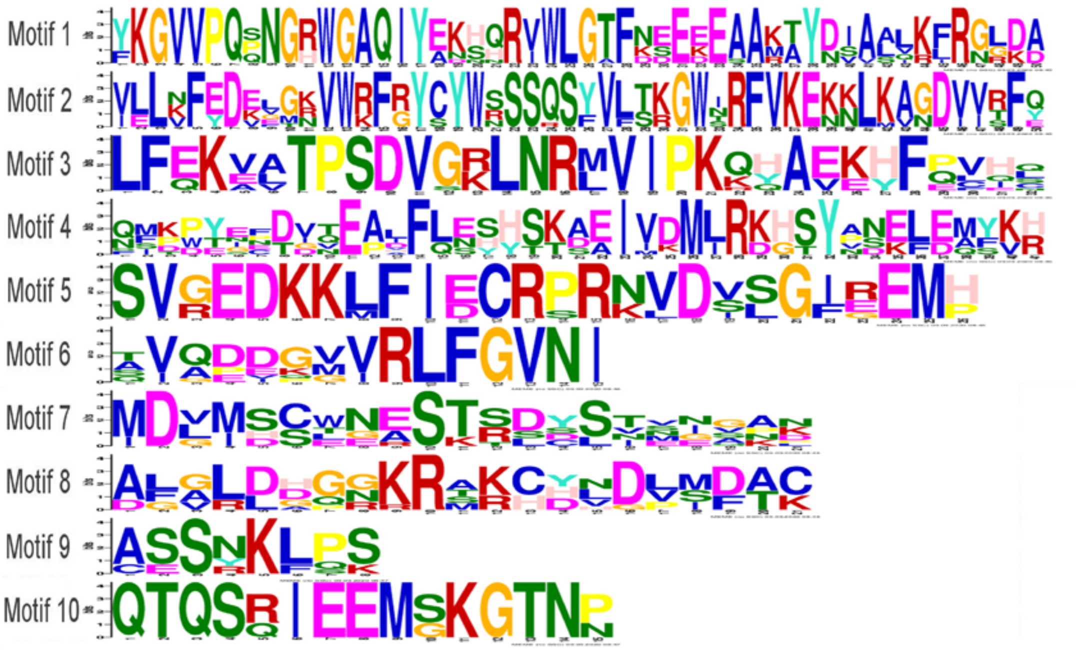

Figure S1. Protein sequences of conserved domains in RAVs

Figure S2

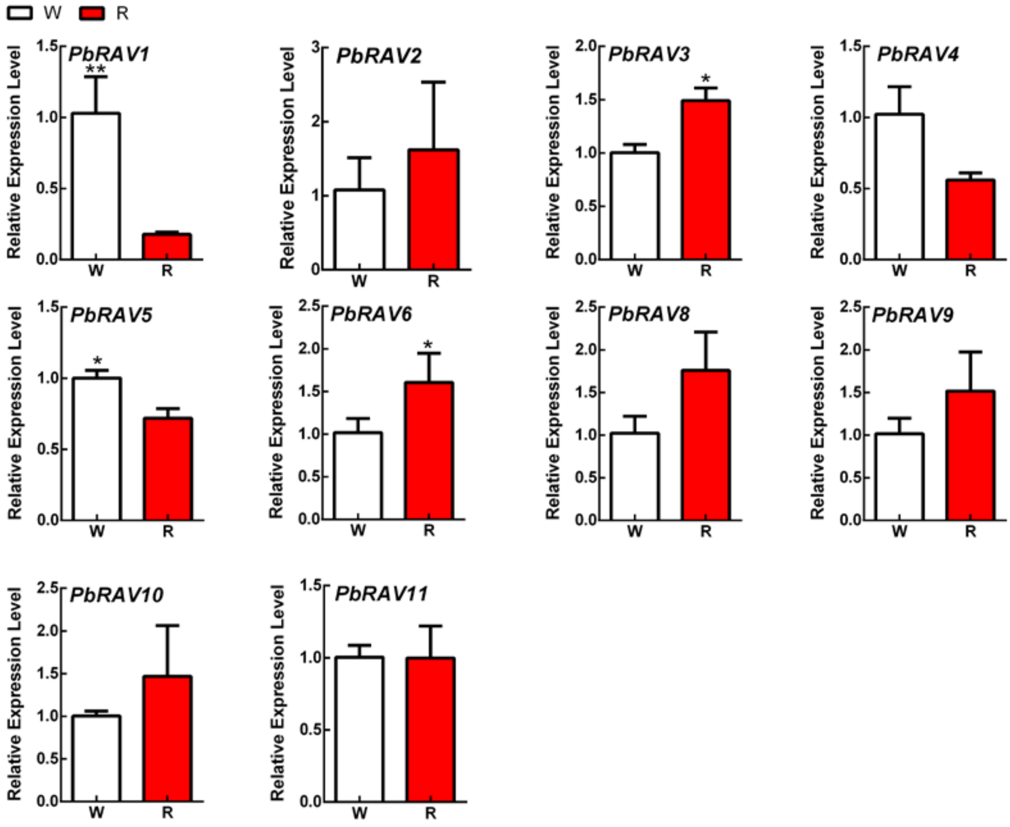

Figure S2. Expression patterns of *PbRAVs* genes between white and red peel. Expression levels of each gene of the white peel were normalized as 1.0. Bars are SD from the mean of three replicates. Asterisks indicate treatment effect using Student's t test (*p < 0.05, **p < 0.01)
