# Supplementary material for: Genome-Wide Analysis of RAV Transcription Factors and Functional Characterization of Anthocyanin-Biosynthesis-Related RAV Genes in Pear
Source: Int J Mol Sci. 2021 May 25;22(11):5567. doi: 10.3390/ijms22115567 (PMC8197526; doi:10.3390/ijms22115567)
Supplement: Supplementary file 1 [file ijms-22-05567-s001.zip › table S.pdf]

Table S1. List of qRT-PCR Primers

Primers	Forward/Reverse	Sequences (5′-3′)
Actin	F	ACAGTGTCTGGATTGGAGGGTC
	R	CATTTGGAGAACTCAGAAGCACT
PbPAL	F	TCTGCCAGGGAAAGATTATCG
	R	TGAAGTTGAATGGAATGGAATGC
Pb4CL	F	CAGTACGTCGATAAGCTCCGA
	R	CTCCGGAGGATCGTCAATCG
PbUFGT	F	CTGGAACCTGAAGTTGTGAATCTG
	R	AGCCACTCTAAGCAACCACTATC
PbGST	F	GGCTGGTGTAAAAGTGCTGG
	R	AACAGTTGACTTGGATCAGGATG
PbMYB10	F	CAGCAGAAGATTTAAGTACGCCATC
	R	TTCTAACAAGGTCTCCCACCAATC
PbRAV1	F	AGAAGAGAGCACAACCAGCG
	R	GTCGGAGTCGAGAATCACGC
PbRAV2	F	AGGCCCTTTTCTTGGAGTCG
	R	CAAGCCGCACAACCATTGTA
PbRAV3	F	GTCGATGTGTCGGGGATCAG
	R	GGCCCGGTAGAAGGTTGTAT
PbRAV4	F	TGTTTGGTTGAGGAGGCCAAT
	R	GGATGCCCTGTGATCTTCACT
PbRAV5	F	TATGTTTGGTTGAGGAGGCCA
	R	CGGCTATGGATGCCCTGTG
PbRAV6	F	GAACAGAGCAAGCGCAACAA
	R	ATCACGAGGCGGTTCAGTTT
PbRAV7	F	ACTTCATCCAAATAAGCGTGCAA
	R	TATTTGTGCACCCCAGTGACC
PbRAV8	F	TCACGGAGGCTCTTTTCTTGG
	R	TCGTACGCTTTCCACCATCG
PbRAV9	F	ATAAGTTGCTCAACGCCGGA
	R	ACCTTCCTACATCGCTTGGC
PbRAV10	F	GGCTGAAATCGTTGACATGCT
	R	ACTTCGTACGCTTTCCACCA
PbRAV11	F	ATAAGTTGCTCAACGCCGGA
	R	ACCTTCCTACATCGCTTGGC
